# Dosing vitamin C in critically ill patients with special attention to renal replacement therapy: a narrative review

**DOI:** 10.1186/s13613-020-0640-6

**Published:** 2020-02-12

**Authors:** Patrick M. Honore, Herbert D. Spapen, Paul Marik, Willem Boer, Heleen Oudemans-van Straaten

**Affiliations:** 1 ICU Dept, Centre Hospitalier Universitaire Brugmann/Brugmann University Hospîtal, Place Van Gehuchtenplein, 4, 1020 Brussels, Belgium; 2 Development, Ageing & Pathology Research Department, Vrije Universiteit Brussel, Brussels, Belgium; 3 Division of Pulmonary and Critical Care Medicine, Eastern Virginia Medical School, 825 Fairfax Av, Suite 410, Norfolk, VA 23507 USA; 4 Dept. of Anesthesiology, Intensive Care Medicine, Emergency Medicine & Pain Medicine, Ziekenhuis Oost-Limburg Genk, Genk, Belgium; 5 Department of Intensive Care Medicine, Amsterdam UMC, Vrije Universiteit Amsterdam, De Boelelaan 1117, 1081 HV Amsterdam, The Netherlands

**Keywords:** Vitamin C, Renal replacement therapy, Sepsis, Burns, Trauma, High-dose vitamin C, Peritoneal dialysis, Prevention of acute kidney injury

## Abstract

**Rationale/methods:**

The primary aim of the present contribution is to find a literature-based agreement on dose adjustments of vitamin C in critically ill patients undergoing renal replacement therapy (RRT).

**Available data/study results:**

Critical illness is frequently accompanied by severe vitamin C deficiency. High-dose supplementation beneficially affects clinical outcome in small cohorts of patients with sepsis, burn injury, and trauma. There are no specific data on clinical outcomes in patients receiving renal replacement therapy (RRT). Vitamin C plasma concentrations in patients on RRT are comparable to critically ill patients not receiving RRT. Vitamin C is cleared from the circulation during RRT at a rate dependent on the plasma concentration, dose and duration of RRT. Sieving coefficient is about 1. While the dose of RRT is lower than normal renal function, tubular reabsorption is absent. Sparse evidence suggests that vitamin C dosing during continuous RRT should not exceed the dose administered to critically ill patients not receiving continuous RRT. Low plasma concentrations are expected during prolonged RRT because of persistent extracorporeal removal, absent renal reabsorption and enhanced metabolic loss due to circuit-induced oxidative stress. A dosage of twice 1 g vitamin C daily may be necessary to achieve normal plasma concentrations during RRT, but more studies are needed. There is no available evidence that high doses of vitamin C administered over a short period can induce oxalate stones or has pro-oxidant effects.

**Conclusions:**

Supplementing vitamin C 1 g twice daily to critically ill patients has a solid pathophysiological rationale and a good safety profile. Patients on RRT probably need similar doses as critically ill patients not receiving RRT. Intravenous vitamin C in a dose of 2 g/day may be necessary to achieve normal plasma concentrations during RRT. However, data on dose adjustment of vitamin C during intermittent or chronic RRT are sparse and require more thorough pharmacokinetic and dose–response studies.

## Rationale for supplementing vitamin C in critically ill patients

Vitamin C has anti-oxidant, anti-inflammatory, and immune-enhancing capacities, and acts as a cofactor for the synthesis of collagen, cortisol, catecholamines, and vasopressin [1]. Plasma vitamin C concentrations frequently flirt with scurvy levels in septic, trauma, and burn patients, after major surgery, and in any condition characterized by overwhelming systemic oxidative and inflammatory stress [2]. Diminished intake, increased consumption, and reduced recycling all contribute to this vitamin C deficiency. In line with its biological modulator functions and continued depletion in severe disease, restoring normal circulating vitamin C levels is thought to improve hemodynamics [3], limit organ failure, and benefit survival of critically ill patients. Promising results have been reported in small cohorts of patients receiving a repletion dose of vitamin C in combination with vitamin E. High-dose intravenous (IV) vitamin C significantly reduced fluid requirements, weight gain, and wound edema and improved renal and pulmonary function in the acute phase after burn injury [4]. Vitamin C infusion, alone [5–7] or in combination with thiamine and hydrocortisone [8], reduced biomarkers of inflammation and endothelial injury and had a positive impact on shock reversal, recovery from organ failure, and survival in severe sepsis and septic shock in a landmark before after trial [8], but not in the most recent vitamins trial which randomized septic shock patients to the above-mentioned cocktail or to hydrocortisone alone [9]. Apart from the hydrocortisone in all control patients, the vitamins trial differs from the before–after trial by a later timing of vitamin C (median more than 24 h after admission), and by less comorbidity. The value of early high-dose IV vitamin C treatment in ischemia/reperfusion injury has been extensively documented in animal experiments [10], but requires confirmation in clinical trials. Altogether, evidence on vitamin C supplementation is still fragmentary or inconclusive and does not support a widespread use in critically ill patients [11, 12]. For instance, regarding the CITRUS-ALI study which is widely quoted and promoted as a negative study; in actuality it is a double-positive study [13]. Indeed, the authors have recalculated the Sequential Organ Failure Assessment (SOFA) score as several errors did occur like for instance imputing the SOFA score prior to death in those patients who died prior to 96 h [5]. This reanalysis demonstrates a significant difference in SOFA scores between the two groups at 96 h and this reanalysis should be published soon [13]. Nevertheless, adjuvant vitamin C therapy holds particular promise in sepsis because of its apparent involvement in sepsis-related pathophysiological processes and the remarkably positive results in small clinical studies. A large number of randomized controlled trials (RCTs) are currently recruiting patients with sepsis and septic shock [14] to assess the benefit of vitamin C alone or in combination with hydrocortisone and thiamine.

## Considerations on dosing of vitamin C in critical illness

The recommended daily dietary intake of vitamin C in healthy individuals is approximately 100 mg and produces plasma vitamin C levels between 60 and 100 µmol/L [15]. Vitamin C dosing in critically ill patients, however, is still an issue under debate. Also, it is unclear whether the dosing strategy should attempt to achieve normal or supraphysiologic (up to 1000 µmol/L) plasma vitamin C levels.

Some items pertaining to vitamin C dosing are known. First, intravenous (IV) dosing is crucial. Enteral uptake is unpredictable and may be seriously limited because the enteral transporter is satiable [1] and gut function often is impaired during critical illness. Second, high vitamin C doses (2–3 g/day) must be administered to restore plasma concentrations to normal [16, 17] and sustained therapy is needed to prevent reoccurrence of hypovitaminosis [17]. Third, a very high dose (100–200 mg/kg/day) is required to obtain supranormal plasma concentrations [4, 17]. Studies which showed beneficial effects on biological and clinical outcome parameters used very high vitamin C doses (66 mg/kg/h or 1584 mg/kg/day) on the first day of admission in burn patients [4] and 3 g, 6 g or 200 mg/kg daily in septic or trauma patients [5–8].

### a. Pharmacokinetics of vitamin C

**Fig. 1:**
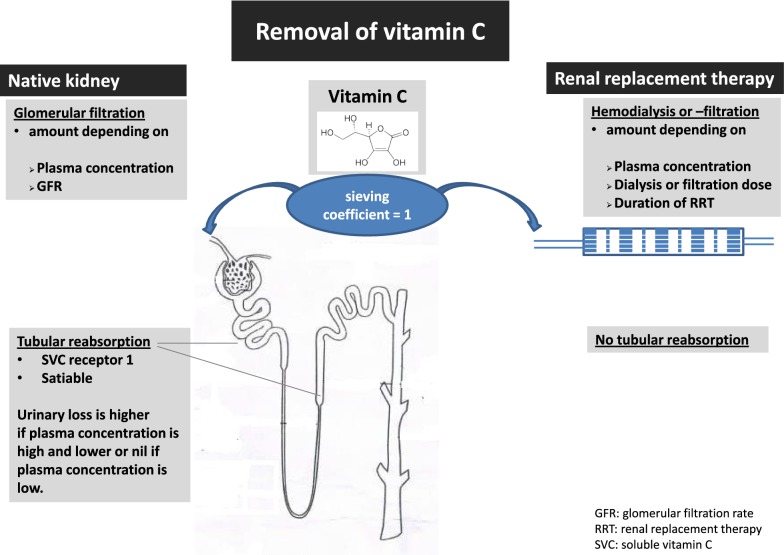
Removal of vitamin C

Vitamin C pharmacokinetics are best described by a two-compartment model with body weight and creatinine clearance as independent covariates [17]. In normal kidneys, vitamin C is filtered in the glomerulus and (partly) reabsorbed in the proximal convoluted tubule and the descending loop of Henle (see Fig. 1). Like enteral absorption, tubular reabsorption is satiable which accounts for a higher loss when plasma concentrations are high. Although the kidneys excrete about half of the administered vitamin C dose, the dose–concentration relationship is linear, implying that an *x*-times higher dose results in *x*-times higher plasma concentration [17]. Plasma concentrations following IV administration are expected to be significantly higher in anuric patients. Nearly half of critically ill patients have or develop acute kidney injury (AKI), and more than 20% need renal replacement therapy (RRT) within the first week of intensive care stay [18].

### b. Pharmacokinetics of vitamin C during RRT

As a small (176 Da) water-soluble molecule, vitamin C concentration in ultrafiltrate or dialysate is equal to plasma (sieving coefficient around 1) [18]. Vitamin C removal during RRT, therefore, depends on the dose and duration of RRT, and on plasma concentrations (see Fig. 1) [19, 20]. During RRT, clearance is less than by glomerular filtration because the dose of standard RRT is lower than normal renal function. However, as vitamin C is not reabsorbed during CRRT, losses persist even in case of overt deficiency due to absent reabsorption. Furthermore, the decline in vitamin C plasma concentration during RRT is higher than expected when based on loss by filtration or dialysis, probably because of consumption related to substantial oxidative stress induced by the extracorporeal circuit [21], which leads to insufficient recycling of the oxidized vitamin C.

### c. Clinical studies on plasma concentrations and loss of vitamin C during RRT

We have summarized the data of the five studies on vitamin C during RRT in Table 1. Vitamin C plasma concentrations in patients on RRT were found to be lower than in healthy controls, but comparable to critically ill patients not receiving RRT (see Table 1). Story et al. reported a daily median loss of 93 (range: 0–372) mg vitamin C in patients on continuous veno-venous hemofiltration (CVVH) [22]. Plasma vitamin C levels were reduced by 50% during a single intermittent hemodialysis session [19, 20]. Morena et al. observed a mean loss of 66 (range 8–230) mg vitamin C per session (200 mg/week) of intermittent chronic hemodiafiltration [19]. Pronounced vitamin C deficiency was reported in 80% of patients receiving continuous RRT (CRRT) for a mean duration of 2 weeks despite a daily IV dose of 500–1.000 mg supplemented for 7 days prior to vitamin C sampling [22].

**Table 1:** Table describing the pertinent studies examining vitamin C dosing in RRT

Author [ref], year	Design and technique used	Sample size	Main findings	Effects on vitamin C levels	Conclusion
Morena [19] 2002	Observational CVVHDF vs. controls	19 HDF patients 1846 healthy controls	Vit C levels significantly lower in HDF patients compared with controls Vit C levels reduced by almost 50% during a HDF session Increased oxidative stress	66 (8–230) mg loss per session of HDF	2/3 loss by diffusion and 1/3 loss by convection
Ferhmann-Ekholm [20] 2008	Observational Low-flux HD vs. on-line HF/HDF	Low-flux HD 15 patients On-line HF/HDF 14 patients	Vit C levels lowered by 51% and 53% in the HD and on-line groups, respectively	Concentrations below reference values in 12/14 patients not receiving vit C supplementation.	Vit C was reduced by both dialysis and filtration treatment
Kamel [21] 2017	Retrospective chart review CRRT	75 patients	80% of patients had below-normal levels of at least 1 macronutrient	Vit C deficiency was identified in 87% (13 of 15)	Vit C deficiency in critically ill patients requiring CRRT was higher than previously reported
Story [22] 1999	Prospective controlled study	8 critically ill patients requiring CVVH 9 critically ill patients not requiring RRT 9 healthy controls	Compared with normal volunteers, critically ill patients on CVVH had significantly lower median blood vit C levels	No differences in serum vit C levels between critically ill patients on CVVH or not on CVVH	Clinical significance of reduced blood concentrations of vit C in critically ill patients and ultrafiltrate losses of vit C remains unclear
Marik [24] 2018	Observational	12 septic shock patients on CRRT receiving 1.5 g vit C 4-times daily	No AKI (*n* = 5) vit C–trough 224 μmol/l peak 543 μmol/l AKI +TCRRT (*n* = 4) vit C–trough 263 μmol/l peak 461 μmol/l CRF + Intermittent HD (*n* = 3) vit C–trough 346 μmol/l peak 914 μmol/l	CVVH at 2 l/h with a sieving coefficient of 1, results in a loss of 1.68 g/vit C/day (200 * 2 * 24) = 9600 μmol/day	6 g vit C daily seems to be adequate in septic shock patients undergoing CRRT

CVVHDF: continuous veno-venous hemodiafiltration; HDF: hemodiafiltration; vit C: vitamin C; HF: hemofiltration, HD: hemodialysis; CRRT: continuous renal replacement therapy; RRT: renal replacement therapy; AKI: acute kidney injury

#### Supplementing vitamin C during RRT by giving 1 g twice daily

Optimal plasma concentrations of vitamin C in critically ill patients on RRT are not known. Furthermore, symptoms of deficiency are difficult to diagnose during critical illness and are probably different from classical scurvy. The dose for supplementation of vitamin C during RRT to prevent scurvy or scurvy-like plasma concentrations of vitamin C is much lower as compared to pharmacological dosing. Apparently, doses of 1000 mg were not enough to avoid low plasma concentrations during RRT in a retrospective study [22]. The plasma vitamin C concentrations of the above described patient on CVVH receiving twice 1 g daily were within the normal range [22], suggesting that a twice daily dose of 1 g vitamin C may be sufficient to maintain normal plasma concentrations during CVVH [23]. However, more pharmacokinetic data are needed for an evidence-based recommendation of vitamin C to prevent deficiency. Plasma vitamin C concentrations in a patient on CVVH receiving 2 g/day were also similar to patients not on CRRT. In this case, the estimated daily effluent loss was 830 mg/day or 41% of the administered dose, which tended to be less than by the native kidney [23].

### d. Pharmacological dosing of vitamin C to manipulate severe oxidative stress during RRT

Dosages of vitamin C should be higher if the goal is to influence redox homeostasis and enzyme function in sepsis and other conditions as severe burns [24]. A recent small case series reported plasma concentrations in CRRT patients supplemented with 6-g vitamin C daily [24]. Trough and peak concentrations were intentionally high (263 and 461 μmol/L resp.), but comparable to patients not receiving CRRT. Estimated effluent losses were 1680 mg/day or 28% of the administered dose. Plasma concentrations in patients with chronic renal insufficiency on intermittent hemodialysis were even higher [24]. Thus, based on the sparse available evidences, vitamin C dosing during CRRT should not exceed the dose administered to critically ill patients not on CRRT. Obviously, more pharmacokinetic data and dose–response studies are needed to settle this issue and randomized controlled trials (RCTs) are needed to evaluate clinical effects in septic AKI needing RRT.

### e. Should a patient on RRT receive IV supplementation if they are on full enteral nutrition?

The answer is yes. Patients on RRT have similar plasma concentrations to critically ill patients not on RRT [23]. Critically ill patients exhibit hypovitaminosis C and vitamin C deficiency despite recommended enteral and parenteral intakes along with nutrition [25, 26]. Based on the scarce clinical data, a twice 1 g dose of intravenous vitamin C is needed to maintain normal plasma concentrations in critically ill patients with or without RRT on full nutrition [23], whereas 1 g/day seems insufficient [21].

### f. Vitamin C dosing during peritoneal dialysis (PD)

Vitamin C deficiency is common in patients undergoing maintenance hemodialysis (MHD) and continuous ambulatory peritoneal dialysis (CAPD). Vitamin C losses are lower in CAPD than in MHD [27]. Patients with chronic kidney disease (CKD) undergoing CAPD, however, are prone to increased oxidative stress (OS) which is associated with enhanced cardiovascular risk, peritoneal membrane changes, and ultrafiltration failure. Supplementation of vitamin C and E in CAPD patients significantly attenuated OS as reflected by an increase in erythrocyte antioxidant enzyme activity and total antioxidant capacity (TAC) and lower MDA and carbonyl compound concentrations [28].

### g. Dosing considerations for patients with renal failure who are not on RRT

Removal of vitamin C by the kidney depends on plasma concentration. In case of hypovitaminosis tubular reabsorption is maximal and removal is minimal while losses are higher when plasma concentrations are high. In patients with severe AKI, plasma concentrations following IV administration are expected to be significantly higher, especially in anuric patients. Thus, dose reduction is needed in patients with renal failure who do not require RRT. Since no guidelines are found in the literature, dosing 1–2 g/day vitamin C dosing seems to be most appropriate to avoid overdosing in this population.

## Renoprotective effects of vitamin C

In a recent animal study, 24 adult male Wistar rats were randomly distributed into three groups: Group I received sevoflurane only, whereas Groups II and III additionally received moderate (150 mg/kg) and high (300 mg/kg) doses of ascorbic acid. The study found a dose-dependent reduction of acute tubular necrosis in the ascorbic acid group [29]. In patients with severe sepsis and septic shock treated with colistin according to a modified pharmacokinetics–pharmacodynamics (PK/PD)-based dosing strategy, Dalfino et al. identified baseline renal impairment and older age as strong predictors of AKI occurrence. Concomitant administration of ascorbic acid markedly reduced AKI risk [30]. In the before–after study of Marik, less patients received RRT for AKI in the group receiving ascorbic acid (in combination with hydrocortisone and thiamine) [8].

## Possible side-effects of treatment with vitamin C

Significant toxicity of high-dose vitamin C has not been reported in published clinical trials. However, in view of a potential increase in vitamin C supplementation in critical illness, vigilance remains imperative.

Some potential side-effects must be emphasized:

### (A) Oxalate stones and nephropathy

High-dose vitamin C increases oxalate excretion [17] and may cause oxalate crystallization, stone formation and nephropathy in susceptible patients. Several cases have been reported in patients receiving vitamin C supplements [31, 32]. Gender is a risk factor for dose-dependent oxalate stone formation [32]. A vitamin C dose above 1000 mg/day was not associated with renal stone formation in women, yet 700 mg/day sufficed to induce stones in men [33]. Apart from primary hyperoxaluria, a rare inborn error of metabolism, risk factors include fat malabsorption due to small bowel resection, inflammatory bowel disease, chronic pancreatitis or gastric bypass (decreasing fecal oxalate excretion), underlying chronic kidney disease, urinary outflow obstruction [34]. However, in a recent prospective case series exploring high-dose vitamin C (up to 100 g IV thrice weekly), no renal stones or kidney injury were reported [35].

### (B) Pro-oxidant effects

Theoretical concerns exist that high-dose vitamin C may exert pro-oxidant effects. By donating an electron during radical scavenging, vitamin C is converted to the ascorbate radical and after a further electron donation to dehydroascorbic acid (DHA). During this process, a more aggressive radical (i.e., superoxide) is converted to the less aggressive ascorbate radical which predominantly reacts with itself, thereby dismutating to DHA and ascorbate [1]. In addition, electrons from ascorbate can reduce copper and iron, and generate superoxide and hydrogen peroxide (H_2_O_2_). This pro-oxidant effect occurs when large doses are infused. Some cancers are susceptible to H_2_O_2_, while human cells are less so possibly because of the large reducing capacity of erythrocytes [1]. Significant toxicity of high-dose vitamin C has not been reported in published clinical trials. However, given that relatively few patients have been enrolled to date, additional side-effects cannot be excluded.

### (C) Incorrect glucose readings

Amongst other factors, high concentration of ascorbic acid interfere with glucose readings from finger stick blood glucose (FSBG) meters [36]. A classic case of marked interference with FSBG readings is due to intravenous ascorbic acid, because the devices recognized ascorbic acid as glucose and erroneously detects hyperglycemia if ascorbic acid levels are high. Factitious hyperglycemia may expose the patient to unwarranted insulin dosing errors. Spectrophotometric methods can be used to avoid unnecessary insulin with the risk of hypoglycemia.

### (D) Hemolysis in G6PD deficiency

Several cases of hemolysis induced by pharmacological doses of IV ascorbic acid (> 60 g) in patients with G6PD deficiency have been published [36]. However, as recently reported low–moderate dose IV vitamin C may be the treatment of choice for drug-induced hemolysis in patients with G6PD deficiency [36]. Extrapolated from in vitro data, a dose of up to 6 g/day is not contraindicated in patients with G6PD deficiency [37]. Higher doses should be avoided in these patients [38].

## Safety

Important side-effects of vitamin C are not reported in any of the mentioned controlled trials, also not in the most recent VITAMIN randomized trial [9]. Furthermore, vitamin C has been evaluated for treating atrial fibrillation in RCTs after cardiac surgery [39]. Meta-analyses also evaluated adverse events. They concluded that vitamin C was safe [40], although meta-analyses upon vitamin C should be taken with caution as recently shown [41].

## Conclusions and future directions

Vitamin C is closely involved in pathophysiological processes related to ischemia–reperfusion, immunomodulation, and inflammation. Critical illness leads to a rapid exhaustion of vitamin C stores. Adjuvant therapy with vitamin C has been shown to mitigate organ injury in burn, sepsis, and post-cardiac surgery patients. The optimal dosing strategy in critically ill patients is unknown and a most effective or specific pathology-related dosing schedule remains to be established. However, at least 2–3 g IV vitamin C must be supplemented daily during the acute phase to normalize plasma concentrations. Preliminary clinical experience suggests that high-dose vitamin C therapy (≥ 6 g daily) in the acute phase is associated with better outcome and no significant toxicity. The promising results of adjuvant high-dose vitamin C alone or in combination with thiamine and hydrocortisone are currently being evaluated in large RCTs.

Scarce available data remain inconclusive regarding dose adjustments during RRT. However, vitamin C dosing during CRRT should probably not exceed the dose administered to critically ill patients not on CRRT, though this hypothesis needs to be confirmed in RCTs. Low plasma concentration can be expected during RRT for a prolonged time period due to persistent extracorporeal removal and metabolic loss due to circuit-induced oxidative stress accentuating the need for further pharmacokinetic and dose–response studies in the setting of CRRT. However, a dosage of about 1 g twice daily may be needed to obtain normal plasma concentration during RRT.

## Data Availability

Not applicable.

## Abbreviations

IV: Intravenous
SOFA Score: Sequential Organ Failure Assessment Score
RCTs: Randomized controlled trials
RRT: Renal replacement therapy
CRRT: Continuous renal replacement therapy
CVVH: Continuous veno-venous hemofiltration
OS: Oxidative stress
DHA: Dehydroascorbic acid
H_2_O_2_: Hydrogen peroxide
MHD: Maintenance hemodialysis
CAPD: Continuous ambulatory peritoneal dialysis
PD: Peritoneal dialysis
TAC: Total antioxidant capacity
CKD: Chronic kidney disease
PK/PD: Pharmacokinetic/pharmacodynamics
